# Edaphic and temporal patterns of *Tuber melanosporum* fruitbody traits and effect of localised peat-based amendment

**DOI:** 10.1038/s41598-020-61274-x

**Published:** 2020-03-10

**Authors:** Sergi Garcia-Barreda, Pedro Marco, María Martín-Santafé, Eva Tejedor-Calvo, Sergio Sánchez

**Affiliations:** 1 Unidad de Recursos Forestales, Centro de Investigación y Tecnología Agroalimentaria de Aragón (CITA), Instituto Agroalimentario de Aragón – IA2 (CITA–Universidad de Zaragoza), Avda. Montañana 930, 50059 Zaragoza, Spain; 2 Centro de Investigación y Experimentación en Truficultura de la Diputación de Huesca (CIET), Polígono Fabardo s/n, 22430 Graus, Spain; 3 Department of Production and Characterization of Novel Foods, Institute of Food Science Research – CIAL (UAM–CSIC), C/Nicolas Cabrera 9, Campus de Cantoblanco, Universidad Autónoma de Madrid, 28049 Madrid, Spain

**Keywords:** Agroecology, Applied microbiology, Fungal ecology

## Abstract

In *Tuber melanosporum* cultivation, fruitbody traits are gaining relevance due to their increasing prominence on prices. We investigated the edaphic and temporal patterns of fruitbody traits and characterised the effect of truffle nests (localised peat-based amendment supplemented with *T. melanosporum* spores) on traits. We monitored fruitbody traits throughout two fruiting seasons in three blocks along a soil gradient. Each trait followed specific edaphic and temporal patterns. The number of fruitbodies per dig and spore maturity followed characteristic within-season trends, whereas fruitbody weight and infestation by truffle beetles were subject to complex interactions among edaphic and temporal variables, suggesting a relevant influence of annual environmental conditions. The application of truffle nests increased fruitbody depth, improved its shape and decreased infestation by truffle beetles. Nests increased the number of fruitbodies per dig, but only in two of the soils, suggesting a relevant role of the bulk soil/substrate interface in fruiting initiation. These results outline a complex scenario, with each trait being differently affected by environmental factors. In this scenario, nests proved to effectively modify several traits, although not always in the desired direction.

## Introduction

The European black truffle (*Tuber melanosporum*) is an ectomycorrhizal fungus highly appreciated in “haute cuisine” due to its organoleptic properties. It grows wild in open oak forests of southern Europe, although nowadays most *T. melanosporum* production is harvested in orchards planted with seedlings previously inoculated in controlled conditions. Truffle cultivation has advanced greatly in recent decades and has expanded worldwide^1^. This is leading to an increased and more stable supply of truffles to markets in recent years, thus encouraging price differentiation by quality^2^. Commercial quality standards are defined by both pre-harvest fruitbody (FB) traits and post-harvest practices^3^. The most important among the former are FB ripeness, gleba colour (associated to spore maturity), abiotic and biotic damages, fresh weight and shape^4^.

Despite recent advances, truffle cultivation is not completely domesticated yet, as the uncertainties around the mating process and the FB formation process remain^5,6^. The environmental mechanisms triggering fruiting and influencing FB development and maturation are still poorly understood^6,7^. Edaphic and climatic factors have a key role in ectomycorrhizal FB yields, phenology and morphology^8–10^. This is particularly likely to apply to truffles, which grow below ground and require several months to develop and ripen^11,12^. Information on the influence of environmental factors on FB traits could help to clarify the ecology of *T. melanosporum* and could be useful for improving productivity and sustainability in truffle cultivation.

Unraveling the linkages between FB traits and edaphoclimatic conditions is difficult because: (1) the long period of formation of truffle FBs hinders the identification of causal relationships, (2) FB traits can be mediated in various ways such as fungal physiology, host physiology or spatio-temporal distribution of resources^9^ and (3) some traits could be linked and trade off with each other. The reproductive investment of black truffle (determined by FB number and size) likely depends on the availability of resources, among which those supplied by the host are fundamental^13^. Water, temperature and their effect on soil O_2_ and CO_2_ diffusion are also key factors for the FB formation in mycorrhizal fungi^9^. On the other hand, maturity and ripeness play an ecologically relevant role in truffle dispersal, which requires completely functional spores as well as the emission of volatile signals attracting animal dispersal vectors. However, the relationship between spore maturity and aroma development is not well understood yet^14^. Attracted by truffle volatiles, truffle beetles (genus *Leiodes*), are among the most frequent consumers of truffle FBs in Spanish *T. melanosporum* orchards, reaching in some cases high levels of FB infestation^15^. Truffle beetles persist as diapausant larvae in the orchard soil from May to September, with adults emerging to copulate and lay eggs near a truffle FB from mid-September to April^16^. One to 1.5 months may be required from the moment adults emerge from the soil until the mycophagous larvae appear^15^.

The uncertainties and gaps in the understanding of FB formation may challenge the productivity and sustainability of truffle cultivation. In many regions, especially the ones in which most orchards are young, the management of truffle orchards is still being adapted to local conditions. Some of the currently applied cultural practices were taken over without change from other regions, whereas other practices have been empirically developed by growers. One of these techniques, the so-called “truffle nests”, “truffle wells” or “truffle traps” (hereafter called “nests”), has widely spread among Spanish growers during the last decade. There are currently over ten commercial substrates and even tractor-implements specifically designed to set up these nests. This technique is an adaptation of an old practice used by wild truffle harvesters, which consisted in the spot application of decomposed organic matter or charcoal hearth soils to amend the soil surrounding a *truffière*^17,18^. One of the innovative aspects of nests application in truffle orchards is the use of peat-based substrates, which clearly differ from mineral soils in many facets: low bulk density, high porosity, high aeration and good drainability, high water retention and easily available water, low thermal conductivity under equal water content and low nutrients content^19–21^. Nests are usually supplemented with truffle spores, which could play a role in reproduction if, as hypothesized by Le Tacon *et al*. (2016)^5^, they could act as male elements in sexual mating. However, despite the widespread use of nests in Spain, their effect on the FB traits has not been characterised yet.

In this study, we aim to investigate the edaphic and temporal patterns of agronomically important *T. melanosporum* FB traits such as weight, maturity, shape and probability of *Leiodes* infestation. We also aim to characterise the effect of nests on FB traits. Truffle growers claim that nests increase truffle quality, although some growers warn on the issues related to the re-wetting problem of peat after drying^22^, while others consider that truffle FBs growing in nests present lower maturity (less melanised spores, lighter-coloured glebae) and ripeness (lower intensity of aroma).

## Results

During fruiting seasons 2016–2017 and 2017–2018, 1865 *T. melanosporum* FBs were harvested in 1212 digs, with nests accounting for 53% of the FBs harvested and 42% of the digs excavated (Table 1). Only four FBs from other *Tuber* species (*Tuber aestivum*) were found.

**Table 1:** Number of *T. melanosporum* fruitbodies harvested and digs excavated.

	Fruitbodies		Digs	
	Bulk soil	Nests	Bulk soil	Nests
Season 2016–2017				
Soil block 1	137	239	112	92
Soil block 2	163	143	129	83
Soil block 3	79	50	71	44
Season 2017–2018				
Soil block 1	178	225	125	94
Soil block 2	166	146	134	91
Soil block 3	149	190	126	111

**Figure 1:**
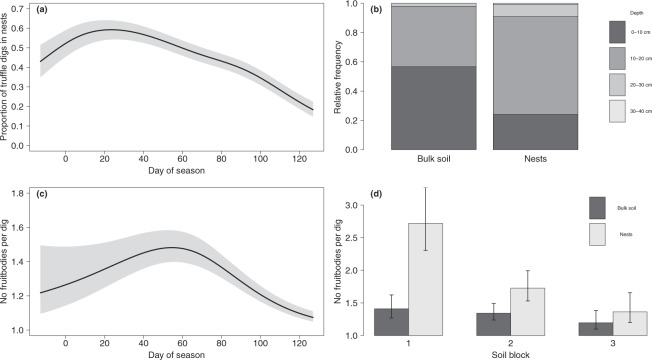
(**a**) Time trend of the proportion (relative frequency) of truffle digs excavated in nests during the fruiting season (n = 1212). (**b**) Depth of truffle digs excavated in bulk soil and nests (n = 1152). (**c**) Time trend of the number of fruitbodies per dig during the fruiting season (n = 1212). (**d**) Effect of the interaction Soil block × Nest on the number of fruitbodies per dig (n = 1212). Fitted GAM predictions, with error bands and bars indicating 95% confidence intervals (α = 0.05). The official harvesting season in Teruel province starts on 15 November (day of season: 0) and ends on 15 March (day of season: 120).

The proportion of digs excavated in nests varied over the fruiting season (P-value adjusted with the Holm-Bonferroni correction for multiple testing, *P*_*Holm*_ < 0.001, Supplementary Table S1), showing a positive trend from its beginning until early December (day of season: 25), after which the relative abundance of digs in nests began to decline (Fig. 1a). Digs excavated in nests remained dominant from mid-November to mid-January (days 0 to 60), falling to their lowest values from mid-February (day 90), a period in which digs excavated in bulk soil became largely dominant. The GAM did not show differences between seasons (*P*_*Holm*_ = 0.36) or among soil blocks (*P*_*Holm*_ = 0.39, Supplementary Table S1).

The depth of the excavated digs ranged from 0–10 to 30–40 cm. Truffles harvested in nests were deeper than those harvested in the bulk soil (*P*_*Holm*_ < 0.001, Supplementary Table S2, Fig. 1b). In 57% of digs excavated in bulk soil FBs were found at depths less than 10 cm, whereas in 67% of excavated nests FBs were found at depths between 10 and 20 cm. Depth did not show any time trend within the fruiting season (*P*_*Holm*_ = 0.71) and was not affected by season or soil block (*P*_*Holm*_ = 0.50 and *P*_*Holm*_ = 0.93 respectively, Supplementary Table S2).

About 74% of the digs excavated presented only one FB, with the remaining digs presenting from two to 17. The number of FBs per dig varied over the fruiting season (*P*_*Holm*_ < 0.001, Supplementary Table S3), with a positive time trend from the beginning of season until early January (day of season: 55), then a decrease until the end of season. The maximum number of FBs per dig was reached from mid-December to the end of January (days 30 to 75), whereas the minimum values happened from late February (day 100, Fig. 1c). The number of FBs per dig was also affected by the interaction between the factors nest and soil block (*P*_*Holm*_ = 0.036, Supplementary Table S3). Nests showed a positive effect in Soil block 1, in which the number of FBs per dig almost doubled with respect to bulk soil; a positive effect in Soil block 2 but only increasing the number of FBs by 30%; and no effect in Soil block 3 (Fig. 1d). No differences were found among bulk soil of the three soil blocks (Fig. 1d). No differences between seasons were found (*P*_*Holm*_ = 0.47).

**Figure 2:**
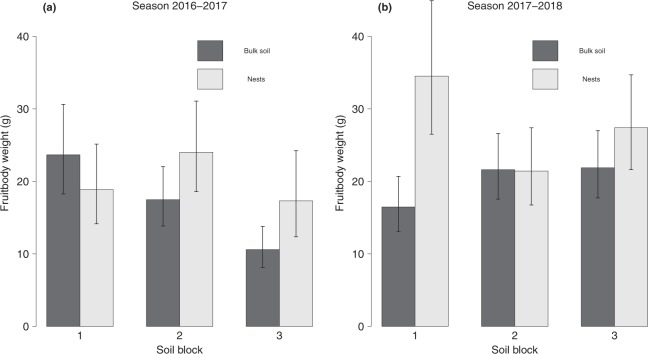
Fruitbody weight (mean predicted values and 95% confidence intervals, α = 0.05) according to the factors soil block and nest, in season 2016–2017 (**a**) and season 2017–2018 (**b**). Only truffles from single-fruitbody digs were considered (n = 604).

The weight of the FBs harvested ranged from 0.2 to 330 g. Fruitbody weight in single-fruitbody digs (digs in which only one FB was found) was affected by the interaction among the factors season, soil block and nest (*P*_*Holm*_ = 0.004, Supplementary Table S4). No differences between nests and the corresponding bulk soil were found for any combination of soil block and season, except for an increased weight in nests from Soil block 1 during season 2017–2018 (Fig. 2). In season 2016–2017, FB weight was higher in bulk soil of Soil block 1 than in bulk soil of Soil block 3, with Soil block 2 in an intermediate situation (Fig. 2). No such differences were found in season 2017–2018. In nests, no differences among soil blocks were found during season 2016–2017 and the same happened in 2017–2018. However, FB weight in nests of Soil block 1 was lower during season 2016–2017 than in 2017–2018 (Fig. 2). The GAM did not show any time trend within the season (*P*_*Holm*_ = 1, Supplementary Table S4).

**Figure 3:**
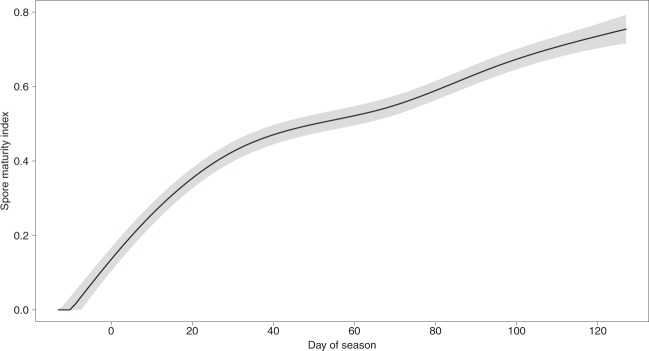
Time trend in the spore maturity index during the fruiting season (n = 576). Fitted GAM predictions, with error band indicating 95% confidence intervals (α = 0.05). The official harvesting season in Teruel province starts on 15 November (day of season: 0) and ends on 15 March (day of season: 120).

The spore maturity index in the measured FBs ranged between 0 and 0.98. Spore maturity in single-fruitbody digs showed a positive trend throughout the fruiting season (*P*_*Holm*_ < 0.001, Supplementary Table S5, Fig. 3). The index increased rapidly from early November until mid-December (days of season −10 to 30) and then more slowly. The GAM did not show differences between seasons (*P*_*Holm*_ = 0.21), among soil blocks (*P*_*Holm*_ = 0.79) or related to the nests (*P*_*Holm*_ = 0.96, Supplementary Table S5).

**Figure 4:**
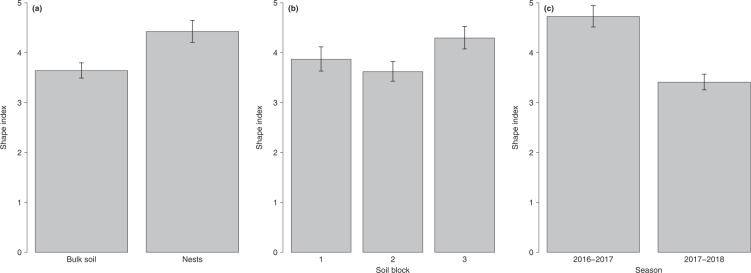
Fruitbody shape index (mean predicted values and 95% confidence intervals, α = 0.05) according to the factors nest (**a**), soil block (**b**) and season (**c**). Only truffles from single-fruitbody digs were considered (n = 891). Shape index ranges from 0 to 8, with higher values indicating more spherical shapes.

The shape index of FBs in single-fruitbody digs was higher in nests than in the bulk soil (*P*_*Holm*_ < 0.001, Supplementary Table S6, Fig. 4a). Soil block 3 showed a higher shape index than Soil block 2, with Soil block 1 in an intermediate situation (*P*_*Holm*_ < 0.001, Supplementary Table S6, Fig. 4b). In season 2016–2017 truffles showed higher shape index than in season 2017–2018 (*P*_*Holm*_ = 0.008, Supplementary Table S6, Fig. 4c). The GAM did not show any time trend within the season (*P*_*Holm*_ = 1, Supplementary Table S6).

The density of the FBs measured ranged between 0.8 and 1.9 g ml^−1^ in FBs weighting more than 10 g, with a few extreme values happening in smaller FBs (0.6–2.9 g ml^−1^). Fruitbody density in single-fruitbody digs was affected by the interaction among season, nest and time (*P*_*Holm*_ < 0.001, Supplementary Table S7). In season 2016–2017, the density in the bulk soil did not show any time trend, whereas in nests it showed a negative trend with pronounced swings (Supplementary Fig. S1). In season 2017–2018 no differences were found between bulk soil and nests in any moment of the season, with both treatments showing a negative time trend. In contrast to bulk soil, in nests the variability in density was particularly high early in the season (Supplementary Fig. S1).

**Figure 5:**
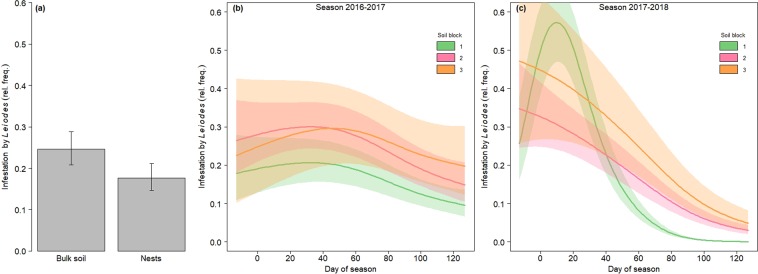
(**a**) Proportion (relative frequency) of fruitbodies infested by *Leiodes* in nests and bulk soil. (**b**) Time trend in the percentage of fruitbodies infested by *Leiodes* according to soil block, during season 2016–2017, and (**c**) season 2017–2018. Fitted GAM predictions, with error bands and bars indicating 95% confidence intervals (n = 1865, α = 0.05). The official harvesting season in Teruel province starts on 15 November (day of season: 0) and ends on 15 March (day of season: 120).

The typical feeding galleries and surface browsing of *Leiodes* larvae and adults were found in 23.1% of the FBs harvested, whereas other abiotic and biotic damages showed much lower incidence: rotting (in 3.1% of FBs), truffle flies from genus *Suillia* (2.9%), surface cracking (1.6%) and freezing (0.5%). The proportion of FBs infested by *Leiodes* was lower in nests than in bulk soil (*P*_*Holm*_ = 0.044, Supplementary Table S8, Fig. 5a). Infestation by *Leiodes* was also affected by the interaction among season, soil block and time (*P*_*Holm*_ < 0.001, Supplementary Table S8). Soil blocks 1 and 2 showed in both seasons a higher *Leiodes* incidence early in the season than at the end of season (Fig. 5b,c). The GAM showed that these time trends were more pronounced in season 2017–2018 than in 2016–2017, with Soil block 3 not showing any time trend in the latter. In season 2017–2018 Soil block 1 presented contrasted differences with the other blocks during part of the season, especially from mid-January (day of season 55, Fig. 5b,c).

## Discussion

This study is the first to provide information on the edaphic and temporal patterns associated with *T. melanosporum* FB traits. Our results indicated that each of the examined traits was differently affected by environmental factors. Furthermore, FB weight and *Leiodes* infestation were subject to interactions among these factors.

The number of FBs per dig varied over the fruiting season. If, as hypothesised by Pacioni *et al*. (2014)^6^, the optimal time for fruiting initiation is characterised by a temperature-moisture window of opportunity, our results could be related to the trends in soil conditions during spring, when fruiting initiation is typically triggered. However, our results could also be explained by time-dependent changes in FB mortality. Intriguingly, the number of FBs per dig did not show differences between seasons or among the bulk soil of the three soil blocks.

Fruitbody weight showed a more complex pattern. The weight of FBs excavated in the bulk soil in season 2016–2017 was influenced by soil properties, with more sandy soils tending to present higher weights. However, considering that in 2017–2018 no differences among soils were found, this effect appears to be strongly dependent on annual environmental conditions, either through their influence on soil microclimate or on host physiology. We hypothesise that interannual differences could be related to soil conditions or tree status during the July–October period, the FB growth stage, during which the more intense development and growth take place^11,23,24^. However, interannual differences in July–October temperature and rainfall in our study site were minor and restricted to the beginning of September (Supplementary Tables S9 and S10, Supplementary Fig. S2). Contrastingly, within each season no differences among the nests of the three blocks were found, suggesting that nests reduced the influence of soil properties.

Spore maturity showed a clear within-season temporal trend, with no apparent influence of the remaining predictors. According to Zarivi *et al*. (2015)^24^, spore melanisation only happens after the FB swelling is completed. Our results show a fast increase in spore maturity from early November to early December that agrees with that view, but in our case maturity continues to increase –much more modestly– throughout the fruiting season. This temporal pattern suggests that climatic conditions and/or fungal physiology rule this process, with a much lower role of soil microclimate variability. Fruitbodies fruiting in late season undergo a much longer period of low soil temperature before ripening than those fruiting in early season.

The predicted spore maturity values reached only around 80% in late season. It must be considered that gleba samples were taken from the outer part. In our experience, in early season spores and glebae are frequently darker in the inner part. Thus, our data could be underestimating spore maturity or overestimating the difference between early and late season. However, this is not likely to affect our conclusions on the remaining predictors.

Fruitbody infestation by *Leiodes* showed a common temporal pattern in most soil-season combinations, with maximum infestation levels in early season (although these peaks were much less marked in season 2016–2017 than in 2017–2018). This pattern could be related to *Leiodes* life cycle, climatic cues or abundance of FBs emitting suitable volatile signals. In a Spanish orchard, three peaks of adult *Leiodes* emergence were found in early, mid and late-season^15^. Our infestation data showed only one early peak, suggesting a relevant role of factors other than abundance of adults.

Beyond this common pattern, *Leiodes* infestation trend showed distinctive features in each season, with more pronounced within-season variation in 2017–2018. In both seasons, the September-February period was relatively dry, with the exception of the very rainy November 2016 (Supplementary Table S10). According to Pérez-Andueza (2015)^15^, adult emergence increases after rainfall, with high soil moisture enhancing the mobility of the insect in the soil. Arzone (1970)^16^ also pointed out that late autumn temperature drop, when accompanied by rainfall, boosts pupation. Our results also revealed that soil properties modulated *Leiodes* infestation trends, with the more clayey soil showing smaller amplitude of within-season variation. Clayey soils generally show lower thermal conductivity^20^ and soil temperature could play a role in adult emergence. Variation among soils and years indicates that soil microenvironment effectively influenced FB infestation by *Leiodes*.

Nests are a common practice in Spanish truffle orchards. Our results demonstrate, for the first time, that nests effectively modify several agronomically important FB traits. Fruitbodies grown in nests showed more spherical shapes and lower probability of infestation by *Leiodes*, thus resulting in improved truffle quality. Shape improvement could be easily explained by the lower and more uniform resistance to penetration of substrate. Lower levels of pest infestation could be related to the light, loose-structured substrate hindering the mobility of adults or impairing the bonding of eggs to soil aggregates^16^. This result concurs with our incidental observation, during the field sampling, that in FBs growing partly in bulk soil and partly in a nest, *Leiodes* galleries were mostly located on the soil side.

Nests clearly increased FB depth. This is very appreciated by Spanish growers, who feel that these FBs are less exposed to abiotic and biotic damages and are more unlikely to suffer from irregular or imperfect ripening. We have not evaluated the incidence of freezing or dryness, but in Australian orchards truffle rot is a particularly alarming problem for shallow FBs^25^.

Nests increased the number of FBs per dig in two of the soil blocks, with the magnitude of this effect being strongly dependent on the soil block. Murat *et al*. (2016)^26^ hypothesised that this increment could be related to spore abundance in the nest if spores were to act as male elements in sexual mating. In our study, spore abundance could be (at least partly) responsible for the differences between bulk soil and nests. However, this factor could hardly explain the different magnitude of the nest effect on each soil block, because the substrate – grinded FBs mix is prepared consistently and systematically for the entire orchard.

Alternatively, the increased number of FBs in nests could be due to enhanced FB survival. Olivier *et al*. (1996)^27^ reported that in June more than ten tiny primordia per square metre of bulk soil can be found, but during the fruiting season harvesters rarely find such clusters of ripe truffles. Considering peat properties and environmental requirements for FB growth, high water availability and aeration in nests could be pivotal in this process.

The higher number of FBs per dig found in nests could also be related to enhanced fruiting initiation. Nest installation creates an abrupt discontinuity in the bulk soil/substrate interface. Pacioni *et al*. (2014)^6^ hypothesised that fruiting initiation is triggered by a change in soil environment, which has already been found for other fungi^9^. In our study, the different magnitude of the nest effect on each soil supports this hypothesis. Moskal *et al*. (2001)^28^ reviewed the effect of peat amendments to soil and concluded that the increase in available water was more marked for sandy than clayey soils.

The question of whether nests increase FB weight remains unclear. Only in one soil-season combination nests affected (positively) FB weight, suggesting complex environmental interactions. This could be related to nests microclimate not always effectively improving FB growth or to a host-mediated effect.

Maturity of single-fruitbody digs was not altered by nests. This indicates that the reported issues with maturity and ripeness in nests, if true, are restricted to multiple-fruitbody digs (digs in which more than one FB was found). When a dog marks a multiple-fruitbody dig, it is only indicating that at least one FB is ripe. Considering that nests increase the number of FBs per dig in some soils, it would be interesting to investigate the presence of unripe truffles in multiple-fruitbody digs.

Fruitbody density showed in early November higher variability in nests than in the bulk soil. October is much warmer than the November-March period (Supplementary Tables S9–S11, Supplementary Fig. S2). This variability could therefore be related to water content of FBs and to the rewetting problem of peat increasing the risk of FB desiccation. Density would thus reflect the microclimate that FBs experience during their last weeks before ripening, highlighting the relevance of irrigation in peat-amended soils. This is supported by the fact that throughout season 2016–2017 density fluctuated in nests while not in the bulk soil. It would merit testing other substrates or soil mixtures that could reduce these issues.

The ratio of digs excavated in nests and bulk soil changed over the fruiting season, indicating a more heavy-tailed distribution over time in nests. Nests seem to particularly promote fruiting in early season. This could be due to the fungus finding conditions encouraging early fruiting initiation, early ripening or increasing the growth rate. Montant and Kulifaj (1990)^7^ found that within irrigated greenhouses (with increased soil temperature in winter-spring and increased soil moisture in summer) much more FBs were harvested in early season, with the typical soil surface cracking linked to accelerated growth of shallow FBs appearing one month earlier^23^. The low thermal conductivity of peat apparently points to a slow increase of temperature in spring and a slow decrease in late autumn. However, soil thermal conductivity is strongly dependent on moisture^20^ and peat shows high water availability at low water potentials.

The changing ratio of digs in nests and bulk soil over the season is likely to result in indirect negative effects of nests on the overall quality of truffles from an orchard, due to the time trends in spore maturity and *Leiodes* infestation. On the positive side, nests promoting fruiting in early December could enhance the profitability of the orchard, due to the peak of black truffle price in the weeks before Christmas.

In conclusion, our results indicate that the edaphic and temporal patterns that drive *T. melanosporum* FB traits are specific for each trait. The number of FBs per dig and the spore maturity showed clear within-season temporal patterns that were consistent from year to year. Contrastingly, FB weight and *Leiodes* infestation were subject to complex interactions among edaphic and temporal variables. In this context, nests effectively increased FB depth, improved shape and decreased *Leiodes* infestation, without decreasing FB maturity in single-fruitbody digs. Our results also gave hints of the issues surrounding peat hydraulic properties. The different effectiveness of nests in increasing the number of FBs among the three soils suggests a relevant role of soil discontinuities in FB initiation. The study shows that nests are a useful tool for advancing in the study of the reproductive biology and ecology of truffles.

## Material and Methods

### Experimental design and data collection

The study was conducted in a truffle orchard in Gúdar-Javalambre county (Teruel province, eastern Spain, 1150 m a. s. l.). The climate is Continental Mediterranean, with a mean annual rainfall of 519 mm and a mean annual temperature of 11.1 °C (Supplementary Table S11). The orchard was planted in 2001 with *Quercus ilex* subsp. *ballota* and *Quercus faginea* seedlings inoculated with *T. melanosporum*. The seedlings were produced in a commercial nursery and controlled by public authorities following the INIA-Aragón method^29^. They were planted at a density of 278 trees ha^−1^ (6 × 6 m), and the two host species were arranged in rows with a 2:1 proportion in the entire experimental site. During the pre-productive stage of the orchard, the soil was tilled once a year, and from the fourth year the trees were annually pruned.

From the moment the orchard began to produce truffles, at the age of 6–10 in all combinations of host tree and soil typology (Supplementary Table S12), a number of cultural practices are annually performed, immediately after the end of fruiting season: tree pruning, soil tillage and nests installation. The soil between tree rows is shallowly tilled with tine harrows (leaving untilled only 1–1.5 m around each tree). Every year, about ten nests are set up around each tree of both host species. The installation of a nest involves digging a tronconical hole about 25 cm deep, filling it with about 1.5 l of a European *Sphagnum* peat-based substrate (Turbatruf ® from Projar: a black peat - white peat - coir - perlite mix 11–5–3–1, with pH raised to 7.5) and re-covering the substrate with soil. Grinded ripe truffle FBs are mixed with the substrate (0.1 g dry fruitbody per liter of substrate), and the mix is thoroughly homogenised before being incorporated into the soil. Nests are systematically arranged at regular intervals along a circumference centred on the tree. Each year a new circumference is set up a little farther, avoiding that the nests radially align with those of previous years. This has generated a quincunx pattern up to a distance of 2 m from the trunk. From April to October, the orchard is irrigated with a sprinkling system during the dry periods with scarce rainfall, following the water-potential criterion of Le Tacon *et al*. (1982)^30^. In season 2017–2018 the orchard was also irrigated from November to January due to low winter precipitations. Truffles are harvested by the owner once a week throughout the fruiting season, from November to March.

Soils with different properties can be found across the 15 ha of the orchard. Three blocks of 0.25 ha were selected in a 300-m-length transect line, with sandy loam Soil block 1 developing on Tertiary siltstones/sandstones, loam/clay loam Soil block 3 on Cretacic clayey limestone and loam Soil block 2 on a mixture of both materials (Supplementary Table S12).

During fruiting seasons 2016–2017 and 2017–2018 each block was repeatedly visited (season 2016–2017: 18/11/2016, 12/12/2016, 10/1/2017, 7/2/2017, 20/2/2017, 7/3/2017 and 22/3/2017; season 2017–2018: 2/11/2017, 16/11/2017, 5/12/2017, 20/12/2017, 10/1/2018, 31/1/2018 and 7/3/2018). In each visit, truffle FBs were harvested with the aid of trained dogs and measured. The weeks in which we did not visit the orchard, truffles were harvested by the owner as usual, once a week.

Each time the dog localised a spot where truffles might be found, the soil was excavated and we recorded whether the truffles grew in bulk soil or in the substrate of nests. Truffles were classified as developed in a nest when the peat kept its characteristic physical structure (without clear signs of degradation) and the nest remained unscathed (without sign of having been disassembled or mixed with soil). In the vast majority of cases, this occurred on 2–4 year-old nests (estimated through the distance to the trunk and the general appearance of the peat, data not shown), as typical in Spanish orchards and consistently with observations in France^26^. We also recorded the number of FBs per dig (each dig corresponding to a dog mark, with all FBs growing together or in very close proximity), depth (of the bottom part of the deepest truffle, at 10 cm intervals) and infestation by *Leiodes* beetles (identification of the distinctive galleries or the browsing in the FB peridium, after gentle brushing).

To avoid confounding the effect of substrate on FB traits with the effect of several truffles growing side by side, the following traits were only measured in single-fruitbody digs: fresh weight (measured to the nearest 0.1 g after gently removing soil and substrate with a brush), shape, density and spore maturity. A shape index was calculated as a combination of (i) sphericity (ratio between the measured maximum and minimum diameter), (ii) lobularity (percentage of the FB surface occupied by lobules), visually estimated, and (iii) average height of lobules in relation to FB size, visually estimated. A categorical classification was defined for each component, thus resulting in a shape index with nine categories (Supplementary Table S13, Supplementary Fig. S3). Fruitbody density was calculated as the ratio between fresh weight and volume (measured by water displacement to the nearest 1 ml). A spore maturity index was calculated as the percentage of asci containing mature (i. e. dark brown and spiny) spores, following Zeppa *et al*. (2002)^31^. Gleba samples reaching 5–10 mm under the peridium were taken with a scalpel, to allow the orchard owner to sell the FBs without price reduction. A minimum of 50 randomly selected asci were counted in each gleba sample to calculate the maturity index.

### Statistical analysis

Generalised additive models (GAMs) were used to analyse the effect of nests on FB traits, the temporal variation of these traits (within a season and among years), the differences in FB traits linked to soil variability and the interactions among all these predictor variables. GAMs allow for non-linear (smooth) effects of predictor variables and different types of error distribution in the response^32^. The time trend within the fruiting season (day of season) was treated as a smoothed predictor variable, whereas the remaining predictors (season, soil block, nest) were included as factors (non-smoothed). A binomial error distribution was used for the proportion of FBs excavated in nests and the proportion of FBs infested by *Leiodes*. A Poisson error distribution was used for FB depth and shape. A negative binomial error distribution was used for the number of FBs per dig, to correct for overdispersion in the Poisson and quasipoisson distributions. A Gaussian (normal) error distribution was used for the FB weight, density and spore maturity. Smooth terms were specified using shrinkage smoothers (cubic regression spline). The interactions of the smooth term with factors were fitted allowing a separate smoother for each level of the factor.

For each FB trait, three GAMs were fitted and the one with the lowest Akaike’s information criterion (AIC) was selected: (i) a model containing only main effects, (ii) a model containing all main effects and two-way interactions, and (iii) a model containing all main effects, two-way and three-way interactions among the variables nest, day of season, season and soil block. These models include every possible interaction because there is no previous research dismissing any of them and their exclusion may alter the results of the statistical analysis. The basis dimension of the smooths was checked and increased if necessary. The fit of the chosen error structure was assessed through overdispersion. Fruitbody weight and density were log transformed to more closely meet the assumptions of normal distribution and constant variance, which were assessed in every normal error distribution model. All analyses were conducted with the package mgcv in R^33^, adjusting P-values with the Holm-Bonferroni correction for multiple testing.

## Supplementary information

Supplementary information.

## Data Availability

The datasets generated and analysed during the current study are not publicly available due to the confidentiality agreement with the orchard owner (the information is considered competitively sensitive from the grower perspective), but are available from the corresponding author on reasonable request.
