# Supplementary material for: Edaphic and temporal patterns of Tuber melanosporum fruitbody traits and effect of localised peat-based amendment
Source: Sci Rep. 2020 Mar 10;10:4422. doi: 10.1038/s41598-020-61274-x (PMC7064507; doi:10.1038/s41598-020-61274-x)

Supplementary information

Edaphic and temporal patterns of *Tuber melanosporum* fruitbody traits and effect of localised peat-based amendment

Sergi Garcia-Barreda, Pedro Marco, María Martín-Santafé, Eva Tejedor-Calvo, Sergio Sánchez

Table S1 Results of the GAM analysis of the proportion of digs in nests
Table S2 Results of the GAM analysis of the FB depth
Table S3 Results of the GAM analysis of the number of FBs per dig
Table S4 Results of the GAM analysis of the FB weight
Table S5 Results of the GAM analysis of the spore maturity
Table S6 Results of the GAM analysis of the shape index
Table S7 Results of the GAM analysis of the FB density
Fig. S1 Time trend in FB density according to the factor nest, in season 2016-2017 and season 2017-2018
Table S8 Results of the GAM analysis of the proportion of FB infested by *Leiodes*
Table S9 Mean monthly temperature for the January 2016-March 2018 period
Table S10 Monthly rainfall for the January 2016-March 2018 period
Fig. S2 Temperature and rainfall in ten-day periods for years 2016 and 2017
Table S11 Mean monthly temperature and rainfall in the study site
Table S12 Soil physicochemical properties of the three soil blocks in the study site
Table S13 Categories defined for the parameters used to estimate the shape index
Fig. S3 Examples of *T. melanosporum* shape characterisation

Table S1 Selected GAM for the proportion of digs in nests (n=1212). Deviance explained: 5.4%. P-values adjusted with Holm-Bonferroni correction for multiple testing.

Terms	Chi-square	P-value [a]	df [b]
Parametric terms			
Season	3.227	0.360	1
Soil block	4.675	0.388	2
Season × Soil block	0.882	0.643	2
Smooth terms			
Time (day of season)	50.877	< 0.001	3.4
Time × Season (2016)	0	1	0
Time × Season (2017)	0	1	0
Time × Soil block (1)	0	1	0
Time × Soil block (2)	0	1	0
Time × Soil block (3)	0	1	0
Time × Season (2016) × Soil block (1)	0	1	0
Time × Season (2017) × Soil block (1)	0	1	0
Time × Season (2016) × Soil block (2)	0	1	0
Time × Season (2017) × Soil block (2)	0.281	1	0.2
Time × Season (2016) × Soil block (3)	3.586	0.216	0.9
Time × Season (2017) × Soil block (3)	4.844	0.090	1.0

[a] Approximate significance for the smooth terms
[b] Estimated degrees of freedom for the smooth terms

Table S2 Selected GAM model for the fruitbody depth (n=1152). Deviance explained: 12.0%. P-values adjusted with Holm-Bonferroni correction for multiple testing.

Terms	Chi-square	P-value [a]	df [b]
Parametric terms			
Season	1.319	0.502	1
Soil block	0.139	0.933	2
Truffle nest	27.166	< 0.001	1
Smooth terms			
Time (day of season)	0.421	0.708	0.3

[a] Approximate significance for the smooth terms
[b] Estimated degrees of freedom for the smooth terms

Table S3 Selected GAM for the number of fruitbodies per dig (n=1212). Deviance explained: 25.1%. P-values adjusted with Holm-Bonferroni correction for multiple testing.

Terms	Chi-square	P-value [a]	df [b]
Parametric terms			
Season	2.537	0.444	1
Soil block	4.737	0.470	2
Truffle nest	40.111	< 0.001	1
Season × Soil block	6.856		2
Season × Truffle nest	5.012	0.200	1
Soil block × Truffle nest	11.819	0.036	2
Season × Soil block × Truffle nest	8.924	0.108	2
Smooth terms			
Time (day of season)	18.088	< 0.001	2.3
Time × Soil block (1)	0	0.970	0
Time × Soil block (2)	0	1	0
Time × Soil block (3)	0	1	0
Time × Season (2016)	0	1	0
Time × Season (2017)	0	1	0
Time × Truffle nest (0)	0	0.743	0
Time × Truffle nest (1)	0	0.965	0
Time × Season (2016) × Truffle nest (0)	0	1	0
Time × Season (2016) × Truffle nest (1)	0	1	0
Time × Season (2017) × Truffle nest (0)	8.059	0.110	2.4
Time × Season (2017) × Truffle nest (1)	0	1	0
Time × Soil block (1) × Truffle nest (0)	5.669	0.099	1.1
Time × Soil block (1) × Truffle nest (1)	0	1	0
Time × Soil block (2) × Truffle nest (0)	0.173	1	0.2
Time × Soil block (2) × Truffle nest (1)	0	1	0
Time × Soil block (3) × Truffle nest (0)	0	1	0
Time × Soil block (3) × Truffle nest (1)	2.187	0.836	0.8
Time × Soil block (1) × Season (2016)	0.703	1	0.5
Time × Soil block (1) × Season (2017)	0	1	0
Time × Soil block (2) × Season (2016)	0	1	0
Time × Soil block (2) × Season (2017)	0	1	0
Time × Soil block (3) × Season (2016)	4.842	0.402	2.0
Time × Soil block (3) × Season (2017)	0	1	0

[a] Approximate significance for the smooth terms

[b] Estimated degrees of freedom for the smooth terms

Table S4 Selected GAM for the fruitbody weight (n=604). Deviance explained: 12.3%. P-values adjusted with Holm-Bonferroni correction for multiple testing.

Terms	F	P-value [a]	df [b]
Parametric terms			
Season	4.5	0.272	1
Soil block	9.366	0.001	2
Truffle nest	1.289	1	1
Season × Soil block	9.777	< 0.001	2
Season × Truffle nest	13.198	0.004	1
Soil block × Truffle nest	3.435	0.297	2
Season × Soil block × Truffle nest	7.95	0.004	2
Smooth terms			
Time (day of season)	0	1	0
Time × Soil block (1)	0	1	0
Time × Soil block (2)	0	1	0
Time × Soil block (3)	0	1	0
Time × Season (2016)	0	1	0
Time × Season (2017)	0.493	0.273	0.8
Time × Truffle nest (0)	0	1	0
Time × Truffle nest (1)	0	0.926	0
Time × Season (2016) × Truffle nest (0)	0	1	0
Time × Season (2016) × Truffle nest (1)	0	1	0
Time × Season (2017) × Truffle nest (0)	0	1	0
Time × Season (2017) × Truffle nest (1)	0	1	0
Time × Soil block (1) × Truffle nest (0)	0.358	0.444	0.8
Time × Soil block (1) × Truffle nest (1)	0	1	0
Time × Soil block (2) × Truffle nest (0)	0	1	0
Time × Soil block (2) × Truffle nest (1)	0.149	0.984	0.5
Time × Soil block (3) × Truffle nest (0)	0	1	0
Time × Soil block (3) × Truffle nest (1)	0.202	0.798	0.6
Time × Soil block (1) × Season (2016)	0	1	0
Time × Soil block (1) × Season (2017)	0	1	0
Time × Soil block (2) × Season (2016)	0.907	0.140	1.2
Time × Soil block (2) × Season (2017)	0	1	0
Time × Soil block (3) × Season (2016)	0	1	0
Time × Soil block (3) × Season (2017)	0	1	0

[a] Approximate significance for the smooth terms
[b] Estimated degrees of freedom for the smooth terms

Table S5 Selected GAM for the spore maturity index (n=576). Deviance explained: 41.4%. P-values adjusted with Holm-Bonferroni correction for multiple testing.

Terms	F	P-value [a]	df [b]
Parametric terms			
Season	5.368	0.210	1
Soil block	0.236	0.790	2
Truffle nest	2.422	0.960	1
Season × Soil block	0.350	1	2
Season × Truffle nest	6.090	0.154	1
Soil block × Truffle nest	0.284	1	2
Season × Soil block × Truffle nest	2.799	0.558	2
Smooth terms			
Time (day of season)	42.941	< 0.001	3.6
Time × Soil block (1)	0	1	0
Time × Soil block (2)	0	1	0
Time × Soil block (3)	0	1	0
Time × Season (2016)	0	1	0
Time × Season (2017)	0	1	0
Time × Truffle nest (0)	0	1	0
Time × Truffle nest (1)	0	1	0
Time × Season (2016) × Truffle nest (0)	0	1	0
Time × Season (2016) × Truffle nest (1)	0	1	0
Time × Season (2017) × Truffle nest (0)	1.288	0.091	1.1
Time × Season (2017) × Truffle nest (1)	0	1	0
Time × Soil block (1) × Truffle nest (0)	0	1	0
Time × Soil block (1) × Truffle nest (1)	0	1	0
Time × Soil block (2) × Truffle nest (0)	0	1	0
Time × Soil block (2) × Truffle nest (1)	0	1	0
Time × Soil block (3) × Truffle nest (0)	0	1	0
Time × Soil block (3) × Truffle nest (1)	0	1	0
Time × Soil block (1) × Season (2016)	0.163	1	0.4
Time × Soil block (1) × Season (2017)	0	1	0
Time × Soil block (2) × Season (2016)	0	1	0
Time × Soil block (2) × Season (2017)	1.735	0.108	2.1
Time × Soil block (3) × Season (2016)	0	1	0
Time × Soil block (3) × Season (2017)	0	1	0

[a] Approximate significance for the smooth terms
[b] Estimated degrees of freedom for the smooth terms

Table S6 Selected GAM for the shape index (n=891). Deviance explained: 14.7%. P-values adjusted with Holm-Bonferroni correction for multiple testing.

Terms	Chi-square	P-value [a]	df [b]
Parametric terms			
Season	10.490	0.008	1
Soil block	27.447	< 0.001	2
Truffle nest	15.407	< 0.001	1
Season × Soil block	4.822	0.450	2
Season × Truffle nest	1.848	0.522	1
Soil block × Truffle nest	4.774	0.368	2
Smooth terms		0	
Time (day of season)	0	1	0
Time × Soil block (1)	1.981	0.516	0.8
Time × Soil block (2)	0	1	0
Time × Soil block (3)	0	1	0
Time × Season (2016)	0	1	0
Time × Season (2017)	2.699	0.343	0.8
Time × Truffle nest (0)	0	0.603	0
Time × Truffle nest (1)	0	0.81	0

[a] Approximate significance for the smooth terms
[b] Estimated degrees of freedom for the smooth terms

Table S7 Selected GAM for the fruitbody density (n=457). Deviance explained: 15.5%. P-values adjusted with Holm-Bonferroni correction for multiple testing.

Terms	F	P-value [a]	df [b]
Parametric terms			
Season	0.986	1	1
Soil block	1.822	1	2
Truffle nest	0.893	1	1
Season × Soil block	0.461	1	2
Season × Truffle nest	0.308	1	1
Soil block × Truffle nest	0.382	1	2
Season × Soil block × Truffle nest	0.146	0.864	2
Smooth terms			
Time (day of season)	0	1	0
Time × Soil block (1)	0	1	0
Time × Soil block (2)	0	1	0
Time × Soil block (3)	0	1	0
Time × Season (2016)	0	1	0
Time × Season (2017)	5.317	< 0.001	1.5
Time × Truffle nest (0)	0	1	0
Time × Truffle nest (1)	0	1	0
Time × Season (2016) × Truffle nest (0)	0	1	0
Time × Season (2016) × Truffle nest (1)	6.124	< 0.001	4.6
Time × Season (2017) × Truffle nest (0)	0	1	0
Time × Season (2017) × Truffle nest (1)	0	1	0
Time × Soil block (1) × Truffle nest (0)	0.210	1	0.8
Time × Soil block (1) × Truffle nest (1)	0	1	0
Time × Soil block (2) × Truffle nest (0)	0	1	0
Time × Soil block (2) × Truffle nest (1)	0	1	0
Time × Soil block (3) × Truffle nest (0)	0	1	0
Time × Soil block (3) × Truffle nest (1)	0	1	0
Time × Soil block (1) × Season (2016)	0	1	0
Time × Soil block (1) × Season (2017)	0	1	0
Time × Soil block (2) × Season (2016)	0	1	0
Time × Soil block (2) × Season (2017)	0.128	1	0.3
Time × Soil block (3) × Season (2016)	0	1	0
Time × Soil block (3) × Season (2017)	0	1	0

[a] Approximate significance for the smooth terms

[b] Estimated degrees of freedom for the smooth terms

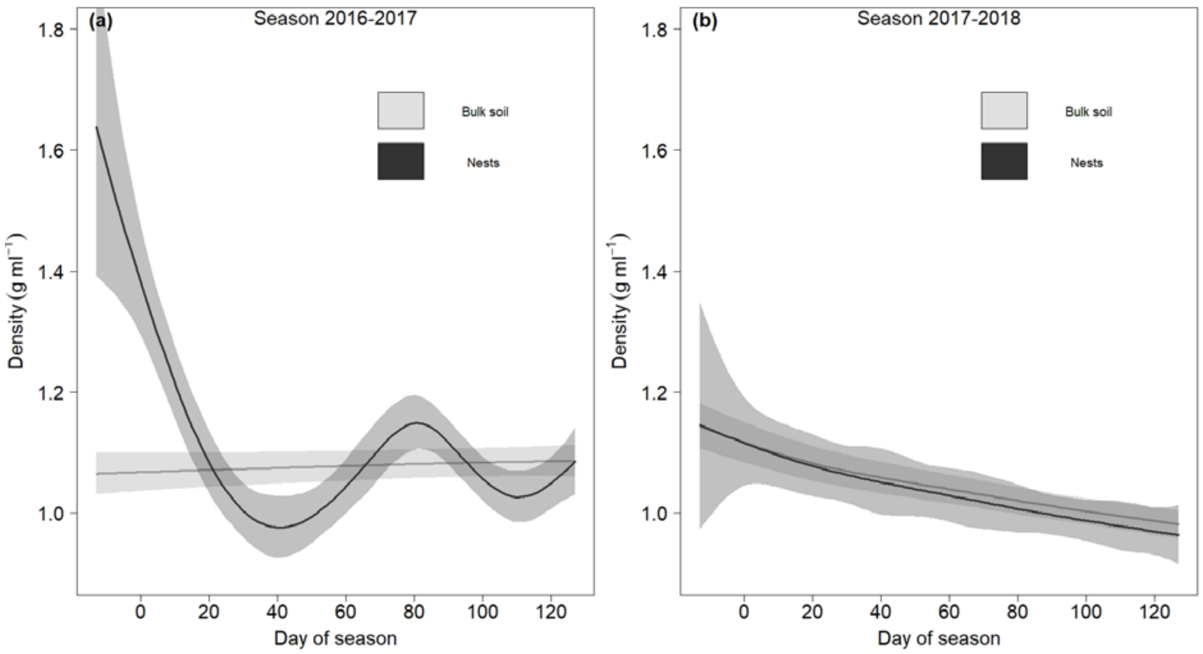

Fig. S1 Time trend in fruitbody density according to the factor nest, in season 2016-2017 (a) and season 2017-2018 (b). Fitted GAM predictions, with error bands indicating 95% confidence intervals (n=457, α = 0.05). The official harvesting season in Teruel province starts on 15 November (day of season: 0) and ends on 15 March (day of season: 120).

Table S8 Selected GAM model for the proportion of fruitbodies infested by *Leiodes* (n=1865). Deviance explained: 9.5%. P-values adjusted with Holm-Bonferroni correction for multiple testing.

Terms	Chi-square	P-value [a]	df [b]
Parametric terms			
Season	4.334	0.370	1
Soil block	1.545	1	2
Truffle nest	8.124	0.044	1
Season × Soil block	1.859	1	2
Season × Truffle nest	2.739	0.784	1
Soil block × Truffle nest	2.652	1	2
Season × Soil block × Truffle nest	5.252	0.648	2
Smooth terms			
Time (day of season)	12.664	0.004	2.1
Time × Soil block (1)	0	1	0
Time × Soil block (2)	0	1	0
Time × Soil block (3)	0	1	0
Time × Season (2016)	0	0.364	0
Time × Season (2017)	11.689	0.002	1.4
Time × Truffle nest (0)	0	0.82	0
Time × Truffle nest (1)	0	0.81	0
Time × Season (2016) × Truffle nest (0)	0	1	0
Time × Season (2016) × Truffle nest (1)	0	1	0
Time × Season (2017) × Truffle nest (0)	0	1	0
Time × Season (2017) × Truffle nest (1)	0	1	0
Time × Soil block (1) × Truffle nest (0)	0	1	0
Time × Soil block (1) × Truffle nest (1)	0	1	0
Time × Soil block (2) × Truffle nest (0)	0	1	0
Time × Soil block (2) × Truffle nest (1)	0	1	0
Time × Soil block (3) × Truffle nest (0)	0	1	0
Time × Soil block (3) × Truffle nest (1)	0	1	0
Time × Soil block (1) × Season (2016)	0	1	0
Time × Soil block (1) × Season (2017)	25.817	< 0.001	3.5
Time × Soil block (2) × Season (2016)	0	1	0
Time × Soil block (2) × Season (2017)	0	1	0
Time × Soil block (3) × Season (2016)	0.667	1	0.4
Time × Soil block (3) × Season (2017)	0	1	0

[a] Approximate significance for the smooth terms
[b] Estimated degrees of freedom for the smooth terms

Table S9 Mean monthly temperature (ºC) for the January 2016 to March 2018 period in the *Confederación Hidrográfica del Júcar* meteorological station located at 5 km from the study site

Month	J	F	M	A	M	J	J	A	S	O	N	D
2016	7.1	6.1	6.4	9.0	12.7	18.4	21.6	20.9	18.0	14.5	7.9	6.5
2017	4.1	6.4	9.3	10.3	14.9	20.0	21.5	21.1	16.8	15.3	8.4	5.1
2018	6.2	3.1	6.3									

Table S10 Monthly rainfall (mm) for the January 2016 to March 2018 period in the *Confederación Hidrográfica del Júcar* meteorological station located at 5 km from the study site

Month	J	F	M	A	M	J	J	A	S	O	N	D
2016	1	25	24	32	46	6	8	24	21	19	89	13
2017	24	13	33	5	25	41	13	35	27	6	4	7
2018	9	27	38									

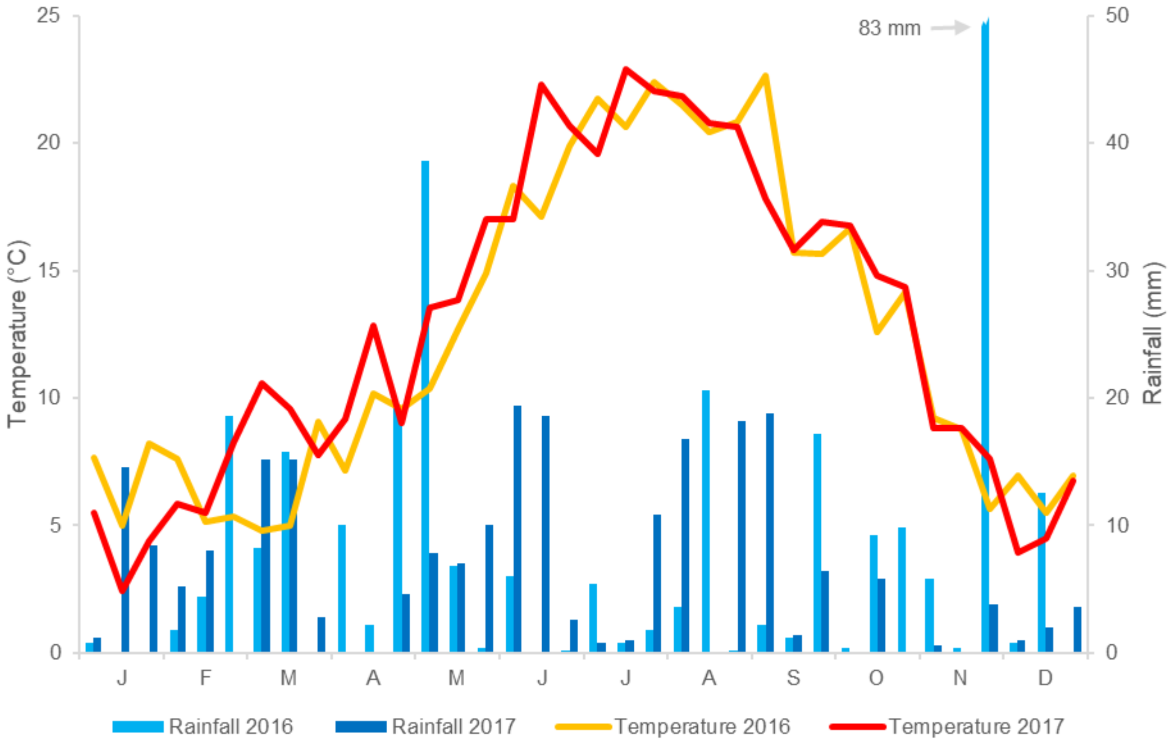

Fig. S2 Average temperature and accumulated rainfall in ten-day periods for years 2016 and 2017. Data from the *Confederación Hidrográfica del Júcar* meteorological station located at 5 km from the study site.

Table S11 Mean monthly temperature (T, in ºC) and rainfall (R, in mm) in the study site from 1981 to 2010 according to the Spanish Climatic Atlas of AEMET (http://agroclimap.aemet.es)

Month	J	F	M	A	M	J	J	A	S	O	N	D
T	3.9	4.9	6.8	8.4	12.1	16.4	20.1	20.0	16.5	11.5	7.2	4.8
R	30	26	27	46	66	52	35	52	51	56	40	38

Table S12 Soil physicochemical properties of the three soil blocks (ST) of the study site. Soils were analysed by *Laboratorio Agroambiental* (Government of Aragón).

	ST 1	ST 2	ST 3
Sand (%)	54.1	46.4	31.7
Silt (%)	27.3	34.5	44.4
Clay (%)	18.6	19.1	23.9
pH, in water 1:2.5	8.3	8.5	8.5
Conductivity (dS m^{-1})	0.4	0.2	0.2
Organic matter (%)	1.2	1.2	1.5
Nitrogen, Kjeldahl (%)	0.07	0.07	0.09
C : N ratio	10.8	9.9	10.0
Phosphorus, Olsen (mg kg^{-1})	5	4	4
Potassium, ammonium acetate extracted (mg kg^{-1})	118	202	246
Calcium carbonate (%)	48	44	51
Active limestone (%)	9.0	6.6	10.5
Magnesium, ammonium acetate extracted (mg kg^{-1})	182	242	314

Table S13 Categories defined for the parameters used to estimate the shape index (Dmin: minimum diameter, Dmax: maximum diameter). The shape index was calculated as the sum of the values assigned to sphericity, lobularity and height of lobules, thus ranging between 0 and 8.

Parameter	Description
Sphericity	
3	Dmin : Dmax ratio higher than 0,85
2	Dmin : Dmax ratio between 0.7 and 0.85
1	Dmin : Dmax ratio between 0.5 and 0.7
0	Dmin : Dmax ratio lower than 0.5
Lobularity (visual estimation)	
3	No lobules
2	Lobules occupying 0-25% of the peridium surface
1	Lobules occupying 25-50% of the peridium surface
0	Lobules occupying more than 50% of the peridium surface
Height of lobules (visual estimation)	
2	No lobules
1	Short lobules
0	Tall lobules (for fruitbodies of about 10 g, lobules taller than 4 mm with respect to the theoretical ellipsoid defined by Dmax and Dmin; for fruitbodies of about 30 g, lobules taller than 6 mm; and for fruitbodies of more than 70 g, lobules taller than 10 mm)

Fig. S3 Examples of *T. melanosporum* shape characterisation (Sph: sphericity, L: lobularity, LH: lobules height). Fruitbodies (a) to (d) depict sphericity categories, fruitbodies (e) to (h) lobularity categories and fruitbodies (i) to (k) lobules height categories. The figure does not keep the proportions among fruitbody images. Note that sphericity categorisation was performed taking into account the three dimensions, 2D images are provided for merely illustrative purposes. In the study site (n=891) the most frequent combinations of shape parameters were (Sph-L-LH): 1-0-0 (11.0%), 2-2-2 (10.1%), 1-1-1 (8.0%), 2-1-1 (7.6%), 2-2-1 (7.3%), 1-2-1 (6.2%) and 1-0-1 (5.7%).

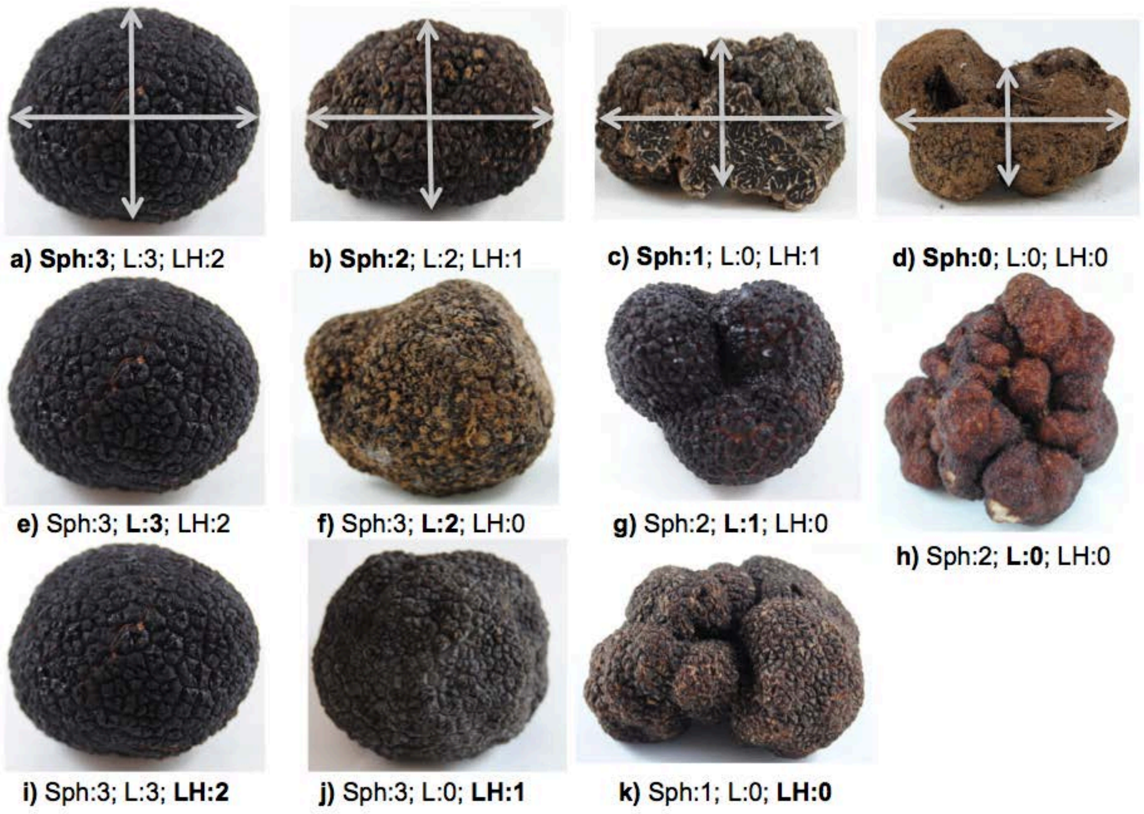

Supplement: Supplementary file 1 — Supplementary information. [file 41598_2020_61274_MOESM1_ESM.pdf]
